# The Treatment of Ascites

**Published:** 1901-06-22

**Authors:** 


### Hospital Clinics and Medical Progress.

### THE TREATMENT OF ASCITES.

At the last meeting of the Royal Medical and Chirurgical Society, Dr. H. Campbell Thomson raised a very important question in regard to the relation between cirrhosis of the liver and the ascites which is so often associated with it. The main point which he made was this, that in association with cirrhosis one may meet with ascites in two forms which differ greatly in regard to prognosis. One form is directly dependent upon the cirrhosis, and ascites of this nature is an almost always fatal condition; a patient suffering from this form of ascites rarely living long enough to survive more than one tapping (the average duration of life, according to Dr. Hale White, after the enlargement of the abdomen is first noticed, being only eight weeks). The ascites which occurs in connection with cirrhosis is not always, however, directly due to this disease, being in many cases the result of chronic peritonitis and perihepatitis, and when so arising it may often be relieved and occasionally may eventually entirely disappear. We have then to recognise that, much as is the case with cirrhosis of the kidney, unless complications arise comparatively early, it is only the later stages of the disease which usually come under notice. Cirrhosis of the liver is indeed a very slowly progressing condition, and may continue for years without showing much sign of its presence. If no accidents happen, it does in the end lead to ascites, and ascites so coming as the direct product of the disease means practically that the end is not far off. But any time in the course of the disease ascites may arise in other ways, chiefly from intercurrent inflammatory conditions affecting the peritoneum, and these forms are often relieved and sometimes cured by tapping, &c., the patient returning again to his former state of latent cirrhosis. Even in such cases, however, the cirrhosis will in time progress, and if the patient lives long enough will ultimately lead to ascites; and when this form, which is directly dependent on the disease, comes on, then the tapping which before had proved so useful becomes of no avail. It must be remembered also that there may be no long interval between the two conditions, the associated ascites often merging into that directly dependent upon the cirrhosis —the form which kills in a few weeks. Turning to the treatment of ascites, Dr. Thomson thought that this could only be effectually done by tapping. Formerly it was advised to put off this measure as late as possible, whereas now it was generally advised to tap early. The more radical operation recently introduced by Morrison and Rutherford was then considered, and it was held that it was in every way a more serious affair than mere tapping, and that if it were to survive it must be shown to possess some distinct advantages over the minor operation. The cases best suited for operation were, it was pointed out, those in which the ascites was only an associated condition; the presence of cirrhosis was not necessarily a contra-indication, but the further advanced the cirrhosis the less the chance must there be of the operation being successful. Certainly if there was good reason to believe that active symptoms of cirrhosis had supervened, and that the ascites was directly dependent upon the cirrhosis, the operation should not be undertaken. Of course, the difficulty is to decide whether or not the cirrhosis is still in its latent condition. In regard to this dependence must be placed to a large extent upon the general condition of the patient. In the discussion, Dr. Hale White insisted on the chronic nature of cirrhosis, *per se*, and said that when investigating a number of cases which proved *post mortem* to have cirrhosis of the liver, he had found that quite half of them had never had any symptom attributable to that condition during life.

